# Device-aided therapies (DATs) in Parkinson’s disease (PD). The DATs-PD GETM Spanish Registry Protocol Study

**DOI:** 10.1371/journal.pone.0316052

**Published:** 2025-03-31

**Authors:** Diego Santos-García, Guillermo González-Ortega, Pilar Sánchez-Alonso, Anna Planas-Ballvé, Rocío García-Ramos, Iria Cabo, Marta Blázquez-Estrada, Álvaro Sánchez-Ferro, DATs-P. D. GETM Spanish Registry Group

**Affiliations:** 1 CHUAC, Complejo Hospitalario Universitario de A. Coruña, A. Coruña, Spain; 2 Grupo de Investigación en Enfermedad de Parkinson y otros Trastornos del Movimiento, INIBIC (Instituto de Investigación Biomédica de A Coruña), A Coruña, Spain; 3 Hospital San Rafael, A. Coruña, Spain; 4 Fundación Degen, A. Coruña, Spain; 5 Hospital Universitario de Móstoles, Madrid, Spain; 6 Hospital Universitario Puerta de Hierro, Madrid, Spain; 7 Complex Hospitalari Moisès Broggi, Sant Joan Despí, Llobregat, Barcelona, Spain; 8 Hospital Universitario Clínico San Carlos, Madrid, Spain; 9 Complejo Hospitalario Universitario de Pontevedra, Pontevedra, Spain; 10 Hospital Universitario Central de Asturias (HUCA), Oviedo, Spain; 11 Hospital Universitario 12 de Octubre, Madrid, Spain; 12 Department of Medicine, Centro de Investigación Biomédica en Red sobre Enfermedades Neurodegenerativas (CIBERNED), Universidad Complutense, Madrid, Spain; National Trauma Research Institute, AUSTRALIA

## Abstract

**Background and objective:**

Device-aided therapies (DATs) are treatments indicated for people with Parkinson´s disease (PwP) experiencing clinical fluctuations that remain suboptimal despite conventional medication. New DATs have recently emerged such as levodopa-entacapone-carbidopa intestinal gel infusion (LECIG) and subcutaneous infusion of foslevodopa/foscarbidopa (fLD/fCD). Understanding the differences between various DATs is essential.

**Patients and Methods:**

We present here the protocol study of the DATs-PD GETM Spanish Registry. This is a descriptive, observational, prospective, multicenter, open study that is proposed as a clinical registry with progressive inclusion of PwP treated with a DAT in daily clinical practice conditions in more 40 centers from Spain for 10 years. The principal aim is to know the type of DAT that PwP in our country (Spain) receive. Specific objectives are to compare the clinical characteristics of the patients, the effectiveness, safety and tolerability, to identify predictors of a good response and to analyze the response by groups (gender, disease duration, phenotype, etc.). There is a baseline visit (V1; indication of the therapy), start visit (V2; initiation of the therapy) and follow-up visits at 6 months ±  3 months (V3_6M) and after this annually ±  3 months for 10 years (V3_12M, V3_24M, etc.).

**Results:**

The registry is on going. The first patient was included on April 10, 2024. Patient recruitment and follow-up will be conducted until 31/DEC/2033. It is estimated that the registry will include a minimum of 3,000 patients.

**Conclusion:**

The present study will help improve the care of PD patients treated with a DAT.

## Introduction

Parkinson´s disease (PD) is the second most common neurodegenerative disease after Alzheimer’s disease. It is characterized by a deficit of dopamine in the striatum and other brain areas, but also of other neurotransmitters such as noradrenaline, acetylcholine or serotonin, which would explain the appearance of motor and non-motor symptoms characteristic of the disease [1]. The diagnosis is made by applying well-defined criteria [2,3] that are based fundamentally on the existence of parkinsonism, the absence of atypical data that suggest an alternative diagnosis (pharmacological, atypical parkinsonism, etc.), and data in favor that suggest PD itself. Among the latter, a determining aspect is the good response to dopaminergic medication, especially levodopa [4]. Its administration compensates for the deficit of dopaminergic stimulation at the level of the postsynaptic receptors, which causes an improvement in symptoms such as tremor, rigidity or bradykinesia, among others. In fact, the absence of a response would go against the diagnosis of PD.

Although the response to dopaminergic medication may be optimal during the initial years, people with PD (PwP) often develop motor and non-motor complications (e.g., motor fluctuations, non-motor fluctuations, dyskinesias, etc.) that affect their quality of life and autonomy [5]. Thus, PwP may only perceive improvement at certain times of the day alternating with disabling OFF episodes where the symptoms reemerge and the control with conventional medication is insufficient [6]. Some PwP may benefit from treatment with a second-line therapy that is an alternative and/or complement to conventional medication that is not sufficient [7]. The proper selection of the candidates is key, and there are different tools for their correct identification [8]. These therapies are commonly known as device-aided therapies (DATs) and although they are more expensive and complex than conventional medication, they have been shown to reduce OFF time, improve non-motor symptoms and the quality of life of PwP [9]. For many years the primary DATs available in numerous countries have been the deep brain stimulation (DBS), continuous subcutaneous apomorphine infusion (CSAI) and levodopa-carbidopa intestinal gel infusion (LCIG) [10]. However, new treatments have recently emerged, such as levodopa-entapacone-carbidopa intestinal gel infusion (LECIG) and subcutaneous infusion of foslevodopa-foscarbidopa (fLD/fCD) [11,12]. Particularly noteworthy is the recent availability of subcutaneous fLD/fCD infusion, to the point that many recently published treatment algorithms are outdated and the possibility of considering the subcutaneous route as the first alternative to the enteral route is being discussed, as it is less invasive when considering infusion therapy [13,14].

Therefore, a completely new scenario is opening up in the treatment of PwP with a DAT and it is essential to know the frequency of the prescriptions, the characteristics of the treated individuals as well as their long-term evolution in relation to clinical changes and complications. This is the reason why, from the Movement Disorders Study Group (Grupo de Estudio de Trastornos de Movimiento [GETM]) of the Spanish Neurological Society (Sociedad Española de Neurología [SEN]), we have launched a prospective registry of PwP treated with a DAT in our country (Spain). The aim of this article is to describe this registry that we have named DATs-PD GETM Spanish Registry.

## Methods/design

**Title of the Project:** Device-aided therapies in Parkinson´s disease GETM Spanish Registry (DATs-PD GETM Spanish Registry).

**Type of study:** Descriptive, observational, prospective, multicenter, open study that is proposed as a clinical registry with progressive inclusion of patients with PwP treated with a DAT in daily clinical practice conditions.

**Promoter and principal coordinator of the project:** Diego Santos García, MD, PhD.

**Coordinating institutions**: “Fundación Degen”, “Grupo de Estudio de Trastornos del Movimiento (GETM)” and “Sociedad Española de Neurología (SEN)”.

**Participating centers in the project:** More than 40 centers from Spain with neurology teams with experience in the management of PD (**Appendix 1**), including the use of DATs.

**Population:** PwP treated with a DAT in Spain from year 2024. The following therapies are included as DATs: DBS; CSAI; LCIG; LECIG; and fLD/fCD subcutaneous infusion. In addition, PwP who are treated with focused ultrasound-guided thermal ablation will also be included. Specifically, the eligibility criteria are: 1) diagnosis of PD according to the MDS criteria [3]; 2) start of treatment with a DAT from January 1, 2024; 3) the patient’s desire to participate on a completely voluntary basis; 4) signing of an informed consent.

**Justification of the project:** 1) there are many DATs currently used to treat PwP; 2) some of them have been introduced very recently and we have no experience; 3) in this context, we must know about the indication preferences in Spanish centers that treat PwP; 4) we must know the characteristics of the individuals treated and the differences between therapies; 4) we must know the effectiveness of DATs and to compare them; 5) we must know the safety and tolerability of DATs and to compare them; 6) it is necessary to define different response profiles or predictors of better or worse outcome; 7) this is an excellent opportunity to be able to develop a registry at this time that collects a lot of information over the long term; 8) It could be a starting point to which centers from other countries can join and develop a broader registry (e.g., European, etc.).

### Objectives

**Principal objective**: to know the type of DAT that PwP in our country receive, treated by expert neurologists in daily clinical practice conditions.

**Specific objectives**: 1) to analyze the sociodemographic and clinical characteristics of PwP treated with a DAT, comparing the different treatments (DBS vs subcutaneous vs enteral treatment); 2) to analyze the effectiveness of different DATs, comparing the different treatments (DBS vs subcutaneous vs enteral treatment); 3) to analyze the safety and tolerability of different DATs, comparing the different treatments; 4) specifically, to analyze the changes experienced by PwP treated with DATs in the perception of their quality of life and autonomy for carrying out daily activities and to compare the different treatments; 5) specifically, to compare the rate of maintenance of the therapy between the different DAT groups; 6) specifically, to compare the dropout rate for each of the therapies and find out the underlying reasons; 7) to find out the reasons for changing from one therapy to another or adding a second DAT to a previous one being received; 8) to analyze the complications in relation to the different DATs and compare them; 9) to define predictors of a favorable response to different DATs; 10) to compare the differences by gender (male vs. female); 11) others that may be raised based on the data collected (e.g., differences by age, disease duration etc.).

### Visits

**Fig 1:**
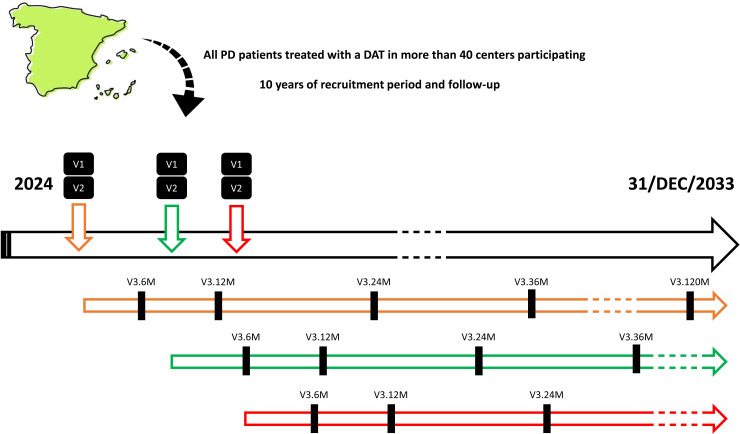
All patients treated with a DAT from 2024 to 31/DEC/2033 in more than 40 participating centers from Spain are included, all of them evaluated by neurologists who are experts in PD. Each color refers to a different patient. There is a baseline visit (V1; indication of the therapy), start visit (V2; initiation of the therapy) and follow-up visits at 6 months (V3_6M) and after this annually (V3_12M, V3_24M, etc.). The window for V2 and V3 is ± 3 months.

The registry includes 3 types of visits: 1) baseline visit (V1), which is when the DAT is decided by the neurologist; 2) start visit (V3), which is when the DAT is initiated by the patient; 3) follow-up visit (V3), with the patient receiving the DAT. The first follow-up visit will be carried out at 6 months + /- 3 months (V3_6M) and then at 1 year + /-3 months and subsequently annually + /- 3 months: 1 year (V3_12M); 2 years (V3_24M); 3 years (V3_36M); 4 years (V3_48M); 5 years (V3_60M); 6 years (V3_72M); 7 years (V3_84M); 8 years (V3_96M); 9 years (V3_108M); 10 years (V3_120M) (**Fig 1**).

Patient inclusion in the registry will span a minimum of 5 years, with the option to extend to 10 years as per the approved protocol. New PwP will be included over a 10-year period, while each participant’s included will be follow-up during this time. The data collection period ranges from January 1, 2024 to December 31, 2033. If a person with PD switches to another DAT or a second DAT is added, the information will be collected. In addition, if a subject drops out of the DAT and does not receive another DAT, data collection will continue within the registry to allow for a comparative arm of untreated patients after a therapeutic failure (something rarely discussed in the literature). Although the registry is prospective, it is planned to include PwP treated in 2024 whose indication for DAT was before this year, as long as the data was properly collected in the medical record. An example is DBS, given that the waiting list from indication to intervention is long in some centers.

### Assessments

The following information will be collected at each visit:

1) Baseline visit (V1): sociodemographic data; data about PD (age onset, disease duration, motor phenotype, etc.); comorbidities; treatments; main reason for therapy indication; levodopa equivalent daily dose (LEDD) [15]; motor symptoms; non-motor symptoms including cognition; the Mini-Mental State Examination (MMSE) [16], Montreal Cognitive Assessment (MoCA) [17] or Parkinson´s Disease Cognitive Rating Scale (PD-CRS) [18] may be used at the discretion of the neurologist in daily clinical practice); Unified Parkinson´s Disease Rating Scale (UPDRS-III) [19] during the OFF state; UPDRS-III during the ON state; Hoehn & Yahr (H&Y) [20] stage in OFF; H&Y stage in ON; MNCD (Motor/Non-motor/Cognition/Dependency) classification (classification; score; stage) [21]; Parkinson’s Disease Questionnaire (PDQ-39) [22]; European Health Interview Survey-Quality of Life 8-item index (EUROHIS-QOL 8-item index) [23]; Schwab and England Activities of Daily Living Scale (ADLS) [24] during the OFF and during the ON state; serum levels of folate, B vitamins (B1, B6, B12) and homocysteine.
2) Start visit (V2): data about the DAT and treatment settings including LEDD.
3) Follow-up visit (V3): data about the DAT and treatment settings including LEDD; comorbidities; treatments; LEDD; motor symptoms; non-motor symptoms; UPDRS-III during the ON state; H&Y stage in ON; MNCD classification (classification; score; stage); Parkinson’s Disease Questionnaire (PDQ-39); EUROHIS-QOL 8-item index; ADLS during the OFF and during the ON state; serum levels of folate, B vitamins (B1, B6, B12) and homocysteine; complications about the DAT and/or other relevant complications.

All information will be collected by expert neurologists in a daily clinical practice setting. A total of 16 and 26 questions collect information on motor symptoms/complications and non-motor symptoms, respectively, which are categorized as absent, mild, moderate, severe, or very severe. The score on the MDS-UPDRS-III scale [25] will be an added option that will be collected in those centers that use it (permission by the MDS was obtained). **Table 1** summarizes the information on the variables that will be collected during the different visits.

**Table 1:** Variables collected in the DAT-PD Spanish Registry according to the type of visit.

V1	V2	V3^*^
Sociodemographic PD history Comorbidities Concomitant treatments LEDD Main reason for DAT PD symptoms Motor symptoms Non-motor symptoms** Motor scales UPDRS-III-OFF UPDRS-III ON H&Y-OFF H&Y-ON Quality of life PDQ-39 EUROHIS-QOL8 Functional dependency ADLS-OFF ADLS-ON Serum biomarkers Folate Vitamin B1 Vitamin B6 Vitamin B12 Homocysteine	Information about DAT Concomitant treatments LEDD	Information about DAT Concomitant treatments LEDD PD symptoms Motor symptoms Non-motor symptoms^**^ Motor scales UPDRS-III-OFF UPDRS-III ON H&Y-OFF H&Y-ON Quality of life PDQ-39 EUROHIS-QOL8 Functional dependency ADLS-OFF ADLS-ON Serum biomarkers Folate Vitamin B1 Vitamin B6 Vitamin B12 Homocysteine Complications Related with DAT Unrelated with DAT

*Information collected for all these visits (with a window of + /- 3 months): 1 year (V3_12M); 2 years (V3_24M); 3 years (V3_36M); 4 years (V3_48M); 5 years (V3_60M); 6 years (V3_72M); 7 years (V3_84M); 8 years (V3_96M); 9 years (V3_108M); 10 years (V3_120M).

**Including cognition; the Mini-Mental State Examination (MMSE), Montreal Cognitive Assessment (MoCA) or Parkinson´s Disease Cognitive Rating Scale (PD-CRS) may be used at the discretion of the neurologist in daily clinical practice).

ADLS, Schwab & England Activities of Daily Living Scale; DAT, device-aided therapy; LEDD, levodopa equivalent daily dose [15]; PD, Parkinson´s disease; PDQ-39, the 39-item Parkinson’s disease Questionnaire; UPDRS, Unified Parkinson´s Disease Rating Scale.

### Data collection and statistical analysis

Data will be collected using REDCap. This is a secure web application for building and managing online surveys and databases. REDCap has been used to date (27/NOV/2024) in 159 countries by more than 3.4 million users. Data collected will be transferred to a statistical package for subsequent analysis. The promoter team of the project will be responsible for study monitoring. The estimate, with the participation of more than 40 centers and a recruitment period of 10 years, is to be able to reach a minimum sample size of 3,000 patients. The pertinent analysis (descriptive, missing data analysis, normality assumptions, univariate, binary logistic regression, multiple linear regression, etc.) will be performed based on the type of objective. In addition, given de complexity of potential analysis including a diversity of variables from different origin and measurement properties, advanced statistical methodology (data mining, machine learning or other artificial intelligence techniques, etc.) could be considered when applicable.

### Standard protocol approvals, registrations, and patient consents

The project will be conducted in accordance with the ICH Good Clinical Practice version 6 Revision 2 standard, the fundamental ethical principles established in the Declaration of Helsinki and the Oviedo Convention, as well as the Spanish legal requirements for biomedical research (Biomedical Research Law 14/2007). The Project has been approved on 02/APR/2024 by the IRB “Comité de Ética de la Investigación Clínica de Galicia from Spain” with code number 2024/109. Written informed consents from all participants in this study will be obtained.

### Study timetable

1) Pre-start-up procedures: until April 2024.
2) First patient included: 10/APR/2024.
3) First analysis to present: 2025 – Q1 (data about PD patients treated in 2024).
4) Proposed specific objectives and others: from 2025 onwards.

### Strengths and limitations of the project

The main strength of the project is the possibility of collecting systematic information on patients treated with DATs over a long follow up period (10 years). Further, there is great interest in the fact that some of these therapies are novel and the management of advanced PD patients is changing because of this and could hence be evaluated. Also, the methodology with prospective follow-up and the collection of well-defined variables (UPDRS, H&Y, PDQ-39, ADLS, etc.) in a clinical practice setting will allow generalizing the findings to other environments. To this end, we also consider in the future increasing the number of centers in Spain, and even the chance of extrapolating the registry to other countries harmonizing the data collected. Other future possibilities are implementing new technologies to monitor the disease and analyze the changes after starting with the DAT and their correlation with the data collected from the registry.

On the other hand and as limitations, unlike a study with a clearly defined protocol in which multiple scales are administered [26], this project is a registry, and limitations in the quantity and quality of the data collected are possible. For example, the number and description of complications may be underestimated. At this moment the project is exciting but there is some residual risk that it will not work properly due to the workload of researchers.

## Discussion

According to the PARADISE study, a non-interventional, cross-sectional, multicenter, national study conducted in the hospital setting and published in 2021, up to 38.2% of people with PD in Spain have advanced PD [27]. The key aspect of identifying advanced PD is to differentiate which PwP could be candidates to receive a DAT. However, only 15.2% of PwP from the advanced PD group from the PARADISE study were receiving some form of therapy for advanced stages of the disease (i.e., DBS, CSAI, LCIG). The most frequent reasons why advanced PwP were not on a DAT were “to be clinically stable” and “option not yet considered”. Similar results were observed in the PROSPECT study [28], in which only 20.9% of PwP (adults with levodopa-responsive PD and inadequately controlled motor symptoms with ≥  2.5-‍hours/day “Off” time, despite trials of available oral/transdermal/sublingual/inhalable medication) initiated a DAT despite the fact that, as expected, DAT vs best medical therapy improved motor symptoms, non-motor symptoms, sleep, quality of life, independence for ADL and caregiver burden. For many years, the options for being treated with a DAT meant choosing between three alternatives: DBS, CSAI, LCIG. However, the scenario is new with the current availability of LECIG and subcutaneous fLD/fCD. In particular, it is of maximum interest to know whether the arrival of fLD/fCD implies a reduction in the indication of other treatments and what are the characteristics of the treated patients as well as their complications in comparison with other DATs, especially enteral therapies [29]. This registry aims to answer this question and many others explained in the objectives. We believe that the information collected in the DATs-PD GETM Spanish Registry will be of great interest in helping to improve the management of PwP treated with a DAT.

In summary, we present here the protocol study of the DATs-PD GETM Spanish Registry, a descriptive, observational, prospective, multicenter, open study that is proposed as a clinical registry with progressive inclusion of PwP treated with a DAT in daily clinical practice conditions in more 40 centers from Spain for 10 years. The present study will help improve the care of PwP treated with a DAT.

## Abbreviations

ADL: activities of daily living
CSAI: continuous subcutaneous apomorphine infusion
DATs: device-aided therapies
DBS: deep brain stimulation
fLD/fCD: foslevodopa-foscarbidopa
H&Y, Hoehn & Yahr; LCIG: levodopa-carbidopa intestinal gel infusion
LECIG: levodopa-entacapone-carbidopa intestinal gel infusion
LEED: levodopa equivalent daily dose
MMSE: Mini-Mental State Examination
MoCA: Montreal Cognitive Assessment
PD: Parkinson´s disease
PD-CRS: Parkinson´s Disease Cognitive Rating Scale
UPDRS: Unified Parkinson´s Disease Rating Scale.

## Appendix 1

Adarmes Gómez AD, Alonso Modino D, Álvarez Sauco M, Aneiros Díaz A, Ávila Rivera A, Baviera-Muñoz R, Belmonte S, Blázquez Estrada M, Caballero Sánchez L, Caballol Pons N, Cabo I, Campins Romeu M, Campolongo A, Cantarero Duque S, Carrillo García F, Casanova Mollá J, Casas Peña E, Castaño García B, Castellano Guerrero AM, Castrillo Sanz A, Cerdán Santacruz DM, Clavero Ibarra P, Cores Bartolomé C, Cots Foraster A, Cubo Delgado E, Delgado Ballestero T, Erdocia Goñi A, Escalante Arroyo S, Escamilla Sevilla F, Espinosa Rosso R, Fanjul Arbos S, Feliz Feliz C, Fernández-Pajarín G, Fernández Revuelta A, Fernández Rodríguez B, Fernández Valle T, Freire Álvarez E, Gamo González E, García Fernández C, García Herruzo A, García Ramos García R, García Ruíz Espiga P, Garrote Espina L, Gil Villar MP, Gómez Esteban JC, Gómez López de San Román C, Gómez Mayordomo V, Gómez Rapela C, González MV, González Ardura J, González Hernández A, González-Ortega G, Guerra Hiraldo JD, Gutiérrez García J, Hernández Vara J, Jesús Maestre S, Kulisevsky Bojarski J, Legarda Ramírez I, López Ariztegui N, López Blanco R, López Dominguez D, López Manzanares L, López Veloso AC, López Valdés E, Lorenzo Barreto P, Lorenzo Brito JM, Lozano D, Macías García D, Madrid Navarro CJ, Martí Andrés G, Martí Martínez S, Martín García R, Martínez Castrillo JC, Martínez-Torres I, Mata Álvarez Santullano M, Mauri Fabrega L, Méndez Guerrero A, Mendoza Rodríguez A, Mir P, Mondragón Rezola E, Monterde Ortega A, Morales Casado MI, Morata-Martínez C, Muñoz Delgado L, Muñoz Ruíz T, Muro I, Novo Ponte S, Ojeda Lepe E, Olivares Romero J, Pagonabarraga J, Pareés Moreno I, Pascual Sedano B, Paz González JM, Peral Quirós A, Pérez Calvo C, Pérez Rangel D, Perona Moratalla A, Planas-Ballvé A, Prendes Fernández P, Quibus Requena L, Rábano Suárez P, Rashid López R, Rebollo Lavado B, Rivero de Aguilar Pensado A, Rojas Pérez E, Ribacoba Díaz C, Romero Fábrega JC, Ruiz López M, Ruíz Martínez J, Samaniego Vinueza LB, San Eufrasio Martínez M, Sánchez Alonso P, Sánchez Ferro A, Sánchez Rodríguez A, Sancho Saldana A, Santos-García D, Sastre Bataller I, Solano Vila B, Solleiro Vidal A, Suárez San Martín E, Tabar Comellas G, Tijero Merino B, Triguero Cueva L, Valero García MF, Valldeoriola Serra F, Vela L, Vinagre Aragón A, Vivas Villacampa L, Vives Pastor B, Yáñez Baña R.

**Table:**

First Name	Last Name	Centre
Diego	Santos García	Complejo Hospitalario Universitario de A Coruña
Jose Manuel	Paz González	Complejo Hospitalario Universitario de A Coruña
Carlos	Cores Bartolomé	Complejo Hospitalario Universitario de A Coruña
Lucia Belen	Samaniego Vinueza	Complejo Hospitalario Universitario de A Coruña
Ángela	Solleiro Vidal***	Complejo Hospitalario Universitario de A Coruña
María	Álvarez Sauco	Hospital General Universitario de Elche
Eric	Freire Álvarez	Hospital General Universitario de Elche
Juan Carlos	Martínez Castrillo	Hospital Universitario Ramón y Cajal
Isabel	Pareés Moreno	Hospital Universitario Ramón y Cajal
Samira	Fanjul Arbos	Hospital Universitario Ramón y Cajal
Ana Belén	Perona Moratalla	Complejo Hospitalario Universitario de Albacete
Inés	Legarda Ramírez	Hospital Universitario Son Espases
Bàrbara	Vives Pastor	Hospital Universitario Son Espases
María Fuensanta	Valero García	Hospital Universitario Son Espases
Esther	Cubo Delgado	Hospital Universitario de Burgos
Nuria	López Ariztegui	Hospital Universitario de Toledo
Maria Isabel	Morales Casado	Hospital Universitario de Toledo
Guillermo	Tabar Comellas	Hospital Universitario de Toledo
Déborah	Alonso Modino	Hospital Universitario de la Candelaria
Jesús Norelis	Lorenzo Brito	Hospital Universitario de la Candelaria
Esther	Rojas Pérez	Hospital Universitario de la Candelaria
Iria	Cabo	Complejo Hospitalario Universitario de Pontevedra
Alejandro	Rivero de Aguilar Pensado	Complejo Hospitalario Universitario de Pontevedra
Jorge	Hernández Vara	Hospital Universitario Vall d´Hebron
Maria Victoria	González	Hospital Universitario Vall d´Hebron
Sara	Belmonte**	Hospital Universitario Vall d´Hebron
Juan Carlos	Romero Fábrega	Hospital Universitario Virgen de las Nieves
Francisco	Escamilla Sevilla	Hospital Universitario Virgen de las Nieves
Lucía	Triguero Cueva	Hospital Universitario Virgen de las Nieves
Carlos Javier	Madrid Navarro	Hospital Universitario Virgen de las Nieves
Rosa	Yáñez Baña	Complejo Hospitalario Universitario de Ourense
Asunción	Ávila Rivera	Complex Hospitalari Moisès Broggi
Núria	Caballol Pons	Complex Hospitalari Moisès Broggi
Anna	Planas-Ballvé	Complex Hospitalari Moisès Broggi
Alejandro	Peral Quirós	Complex Hospitalari Moisès Broggi
Dolors	Lozano	Complex Hospitalari Moisès Broggi
Álvaro	Sánchez Ferro	Hospital 12 de Octubre
Pablo	Rábano Suárez	Hospital 12 de Octubre
Antonio	Méndez Guerrero	Hospital 12 de Octubre
Daniel	Pérez Rangel	Hospital 12 de Octubre
Frances	Valldeoriola Serra	Hospital Clínic
Rocío	García Ramos	Hospital Clínico Universitario San Carlos
Ana	Fernández Revuelta	Hospital Clínico Universitario San Carlos
Eva	López Valdés	Hospital Clínico Universitario San Carlos
Carmen	Ribacoba Díaz	Hospital Clínico Universitario San Carlos
Irene	Martínez-Torres	Hospital Universitario la Fe
Carlos	Morata-Martínez	Hospital Universitario la Fe
Raquel	Baviera-Muñoz	Hospital Universitario la Fe
Marina	Campins Romeu	Hospital Universitario la Fe
Isabel	Sastre Bataller	Hospital Universitario la Fe
Pilar	Sánchez Alonso	Hospital Puerta de Hierro
Sabela	Novo Ponte	Hospital Puerta de Hierro
Elisa	Gamo González	Hospital Puerta de Hierro
Raquel	Martín García	Hospital Puerta de Hierro
Pablo	Mir	Hospital Universitario Virgen del Rocío
Laura	Muñoz Delgado	Hospital Universitario Virgen del Rocío
Astrid Daniela	Adarmes Gómez	Hospital Universitario Virgen del Rocío
Elena	Ojeda Lepe	Hospital Universitario Virgen del Rocío
Silvia	Jesús Maestre	Hospital Universitario Virgen del Rocío
Daniel	Macías García	Hospital Universitario Virgen del Rocío
Fátima	Carrillo García	Hospital Universitario Virgen del Rocío
Ana María	Castellano Guerrero***	Hospital Universitario Virgen del Rocío
Manuela	San Eufrasio Martínez***	Hospital Universitario Virgen del Rocío
Cristina	Pérez Calvo***	Hospital Universitario Virgen del Rocío
Andrés	García Herruzo	Hospital Universitario Virgen del Rocío
Cristina	Gómez Rapela*****	Hospital Universitario Virgen del Rocío
Lorena	Garrote Espina*****	Hospital Universitario Virgen del Rocío
Marta	Blázquez Estrada	Hospital Universitario Central de Asturias
Esther	Suárez San Martín	Hospital Universitario Central de Asturias
Ciara	García Fernández	Hospital Universitario Central de Asturias
Patricia	Prendes Fernández****	Hospital Universitario Central de Asturias
Pedro	García Ruíz Espiga	Hospital Fundación Jiménez Díaz
Cici	Feliz Feliz	Hospital Fundación Jiménez Díaz
Raúl	Espinosa Rosso	Hospital Universitario de Jerez
Lara	Mauri Fabrega	Hospital Universitario de Jerez
Jaime	Kulisevsky Bojarski	Hospital Sant Pau de Barcelona
Berta	Pascual Sedano	Hospital Sant Pau de Barcelona
Javier	Pagonabarraga	Hospital Sant Pau de Barcelona
Antonia	Campolongo**	Hospital Sant Pau de Barcelona
Marina	Mata Álvarez-Santullano	Hospital Universitario Infanta Sofía
Juan Carlos	Gómez Esteban	Hospital de Cruces
Tamara	Fernández Valle	Hospital de Cruces
Marta	Ruiz López	Hospital de Cruces
Beatriz	Tijero Merino	Hospital de Cruces
Lydia	López Manzanares	Hospital Universitario La Princesa
Inés	Muro	Hospital Universitario La Princesa
Elena	Casas Peña	Hospital Universitario La Princesa
Pablo	Lorenzo Barreto	Hospital Universitario La Princesa
Maria Pilar	Gil Villar	Hospital Universitari Arnau de Vilanova
Agustín	Sancho Saldana	Hospital Universitari Arnau de Vilanova
Laura	Quibus Requena	Hospital Universitari Arnau de Vilanova
Jesús	Olivares Romero	Hospital Universitario de Torrecárdenas
Belén	Rebollo Lavado	Hospital Universitario de Torrecárdenas
Lucía	Triguero Cueva*	Hospital Universitario de Torrecárdenas
Víctor	Gómez Mayordomo	Hospitales Vithas
Berta	Solano Vila	Hospital Josep Trueta de Girona y Hospital Santa Caterina de Salt
Anna	Cots Foraster	Hospital Josep Trueta de Girona y Hospital Santa Caterina de Salt
Daniel	López Dominguez	Hospital Josep Trueta de Girona y Hospital Santa Caterina de Salt
Lilian	Vivas Villacampa	Hospital Josep Trueta de Girona y Hospital Santa Caterina de Salt
Jessica	González Ardura	Hospital de Cabueñes
Belén	Castaño García	Hospital de Cabueñes
Antonio	Sánchez Rodríguez	Hospital de Cabueñes
Sonia	Escalante Arroyo	Hospital Virgen de la Cinta
Tania	Delgado Ballestero	Parc Taulí
Débora María	Cerdán Santacruz	Hospital General de Segovia
Amelia	Mendoza Rodríguez	Hospital General de Segovia
Ana	Castrillo Sanz	Hospital General de Segovia
Lorena	Caballero Sánchez	Hospital General de Segovia
Claudia	Gómez López de San Román	Hospital General de Segovia
Gustavo	Fernández-Pajarín	Complejo Hospitalario Universitario de Santiago de Compostela
Jordi	Casanova Mollá	Hospital Universitario Sant Joan de Reus
Ángela	Monterde Ortega	Hospital Universitario Joan XXIII
Susana	Cantarero Duque	Hospital Universitario de Móstoles
Guillermo	González-Ortega	Hospital Universitario de Móstoles
Beatriz	Fernández Rodríguez	Hospital Regional Universitario de Málaga
Teresa	Muñoz Ruíz	Hospital Regional Universitario de Málaga
Gloria	Martí Andrés	Hospital Universitario de Navarra, Pamplona, Navarra
Pedro	Clavero Ibarra	Hospital Universitario de Navarra, Pamplona, Navarra
Amaia	Erdocia Goñi	Hospital Universitario de Navarra, Pamplona, Navarra
Ana Carolina	López Veloso	Hospital Universitario Doctor Negrín, Las Palmas de Gran Canaria
Ayoze Nauzet	González Hernández	Hospital Universitario Doctor Negrín, Las Palmas de Gran Canaria
Juan Diego	Guerra Hiraldo	Hospital Universitario Doctor Negrín, Las Palmas de Gran Canaria
Raúl	Rashid López	Hospital Puerta del Mar, Cádiz
Raúl	Espinosa Rosso	Hospital Puerta del Mar, Cádiz
Silvia	Martí Martínez	Hospital General Universitario Alicante
Ángel	Aneiros Díaz	Complejo Hospitalario Universitario de Ferrol
Lydia	Vela	Hospital Fundación de Alcorcón
Javier	Ruíz Martínez	Hospital Universitario Donostia
Elisabet	Mondragón Rezola	Hospital Universitario Donostia
Ana	Vinagre Aragón	Hospital Universitario Donostia
Javier	Gutiérrez García	Complejo Hospitalario Clínico San Cecilio, Granada
Roberto	López Blanco	Hospital Universitario Severo Ochoa, Leganés, Madrid

The researchers are listed by center in chronological order according to the date they confirmed their participation in the project.

* This investigator works at 2 different centers.

All investigators are neurologists except those ones with the symbol:

** SN (specialized nurse);

*** graduate in Biology (she will be responsible for remote data monitoring);

**** neuropsychologist;

***** , study coordinator.
