# Supplementary material for: Device-aided therapies (DATs) in Parkinson’s disease (PD). The DATs-PD GETM Spanish Registry Protocol Study
Source: PLoS One. 2025 Mar 31;20(3):e0316052. doi: 10.1371/journal.pone.0316052 (PMC11957350; doi:10.1371/journal.pone.0316052)
Supplement: Attachment — Submitted filename: Reply.to.reviewers.PLOS.One.25.ENE.2025.docx [file pone.0316052.s002.docx]

A Coruña, 25.JAN.2025

Dear Editor,

We are submitting a revised version (R1) of the manuscript PONE-D-24-54866] entitled **“Device-aided therapies (DATs) in Parkinson´s disease (PD). The DATs-PD GETM Spanish Registry Protocol Study”.**

Thank you for your letter of 21 January, 2025. We appreciate all your comments and suggestions as well those from the reviewer's of our manuscript: we have modified the manuscript as their suggestion and a point-by-point response to the reviewers’ comments is enclosed. Moreover, Appendix 1 has been updated. We would be pleased to provide additional information if requested or to further modify the text.

Correspondence will be send to the following address: Dr. Diego Santos-García, Department of Neurology – CHUAC (Complejo Hospitalario Universitario de A Coruña), c/ As Xubias 84, 15006, A Coruña, Spain; e-mail: [diegosangar@yahoo.es](mailto:diegosangar@yahoo.es).

We look forward to hearing from you. Thank you for your attention to our submission.

Sincerely yours,

Diego Santos García

Reviewer #1: In this paper the authors present the protocol for a register for patients with PD on DAT. This is very important as there is a clear lack of comparative, longitudinal studies between different treatment options of adavnced PD.

The forst objective of the registry would be to:

1) to analyze the sociodemographic and clinical characteristics of PwP

treated with a DAT, comparing the different treatments (DBS vs subcutaneous vs enteral

treatment). It is not clear what subcutaneous (subcutavneous apomorphine pump and foslevodopa/foscarbidopa pump) and eneteral (LCIG, LECIG) DAT options would be taken into account. This needs to be sepcified.

**AUTHORS – Thank you very much for your comment. That is indeed the first objective into the specific objectives: “1) to analyze the sociodemographic and clinical characteristics of PwP treated with a DAT, comparing the different treatments (DBS vs subcutaneous vs enteral treatment)”. The reason for including all therapies has been justified in the introduction.**

There is an important objective missing - that is to define predictors of a favourable response to different DATs.

**AUTHORS – Thank you very much for your comment. We agree with you. This objective has been added. Many thanks again.**

Could the authors forsee how long the study will last for?

"The first follow-up visit will be carried out at 6

months +/- 3 months (V3_6M) and then at 1 year +/-3 months and subsequently annually +/- 3

months: 1 year (V3_12M); 2 years (V3_24M); 3 years (V3_36M); 4 years (V3_48M); 5 years

(V3_60M); etc." ETc is not good enough. Please, specifiy - indefenete, or for at least 10 years, or up to 20 years depending on funding...

**AUTHORS – Thank you very much for your comment. This aspect is explained in the protocol: “The period of inclusion of patients in the registry will be a minimum of 5 years, extendable to 10 years according to the approved protocol. New PwP will be included over a 10-year period, while each participant's included will be follow-up during this time. The data collection period ranges from January 1, 2024 to December 31, 2033”. As you suggest, we change the sentence and all detail data about the visits: “The first follow-up visit will be carried out at 6 months +/- 3 months (V3_6M) and then at 1 year +/-3 months and subsequently annually +/- 3 months: 1 year (V3_12M); 2 years (V3_24M); 3 years (V3_36M); 4 years (V3_48M); 5 years (V3_60M); 6 years (V3_72M); 7 years (V3_84M); 8 years (V3_96M); 9 years (V3_108M); 10 years (V3_120M) (Figure 1)”.**

Also, Table 1 is confusing - at present, it seems there are going to be three visits only!

**AUTHORS – Thank you very much for your comment. This point has been clarified in the table. Please, see the symbol * and the explanation: “Information collected for all these visits (with a window of +/- 3 months): 1 year (V3_12M); 2 years (V3_24M); 3 years (V3_36M); 4 years (V3_48M); 5 years (V3_60M); 6 years (V3_72M); 7 years (V3_84M); 8 years (V3_96M); 9 years (V3_108M); 10 years (V3_120M)”.**

Figure 1. Please change 31/Dic/2033 to 31/Dec/2033 in

**AUTHORS – Thank you very much for your comment. It has been corrected.**

The ABtract needs to be imporved - it does not reflect the exat content of the paper. For example, lines 58-60 in the Abstract - It is not clear of wheterer the follow-up would last for 2, 3 years, or longer as explained later on in the paper.

**AUTHORS – Thank you very much for your comment. In the abstract, the methods about the project (main and specific objectives, types of visits, start and end of recruitment, etc.) are explained. Since there is a 250 word limit we have added at the end of the methods section as you indicate "for 10 years". Other sentences have been modified.**

Please correct typos - for example Abril to April (line 9, page 1) in the Abstract.

Instead of People with Parkinson's disease (PwP) might be Subject(s) with Parkinson's disease (SPD). Xonsider change it.

**AUTHORS – Thank you very much for your comment. Typos have been corrected. We appreciate your suggestion but if you don´t mind we prefer to maintain the term PwP because it has been extensively used in the literature compared with the other term (**[**https://pubmed.ncbi.nlm.nih.gov/?term=PwP+people+with+parkinson&sort=date**](https://pubmed.ncbi.nlm.nih.gov/?term=PwP+people+with+parkinson&sort=date)**).**

Reviewer #2: The present protocol has been drafted to inform about clinical reasoning, management, and possible beneficial as well as side effects of newly-introduced device-aided therapies, such as levodopa-entacapone-carbidopa intestinal gel infusion (LECIG) and subcutaneous infusion of foslevodopa/foscarbidopa (fLD/fCD).

The protocol looks good and I have some minor suggestions:

- I would avoid using the term "DAT" as it is misleading (too similar to the DaT SPECT Scan). I would rather use "DT" or avoid the abbreviated form.

**AUTHORS – Thank you for your comment. I understand your suggestion. We have decided to use DAT because is the abbreviation used in the literature of device-aided therapy. In all the recent articles about this topic, DAT is always used (**[**https://pubmed.ncbi.nlm.nih.gov/?term=DATs+device-aided+therapies**](https://pubmed.ncbi.nlm.nih.gov/?term=DATs+device-aided+therapies)**). This is why we understand the importance of using the term DAT to ensure consistency in the literature and facilitate the dissemination of the work.**

- In the introduction, I would avoid using the term "parkinson plus syndrome" as it is obsolete. I would rather use "atypical parkinsonism" instead.

**AUTHORS – Thank you for your comment. The term has been changed.**

- In the Methods, Line 166, I would specify at point 1: "Clinical diagnosis of PD according to the MDS criteria". Please, add a citation here.

**AUTHORS – Thank you for your comment. This is the reference 3. It has been added: “1) diagnosis of PD according to the MDS criteria [3];”.**

- Assessments: Are the authors planning to collect yearly MoCA and/or any cognitive screening tests? I believe this is highly informative. Also, authors use both the terms UPDRS and MDS-UPDRS. I hope they are going to use the MDS-UPDRS scale. Please, clarify. Finally, "MNCD" and "EUROHIS-QOL" should be spelled out. Please, spell out LEDD and explain the methods of calculation (e.g., Tomlinson 2010 or more recent amendments).

**AUTHORS – Thank you for your comment.** **In patients with cognitive impairment, the MMSE, MOCA or PD-CRS may be used at the discretion of the neurologist in daily clinical practice, but due to problems with sufficient time to collect the information, it will not be performed in a regulated manner in all patients. We have added this information: “non-motor symptoms including cognition; the Mini-Mental State Examination (MMSE) [16], Montreal Cognitive Assessment (MoCA) [17] or Parkinson´s Disease Cognitive Rating Scale (PD-CRS) [18] may be used at the discretion of the neurologist in daily clinical practice)”. The protocol explains that the UPDRS-III will be used and that, in addition, in some centers that use it regularly in clinical practice, information from the MDS-UPDRS-III could also be collected. In line 219, the term LEDD appears spelt out and there is a reference about how to calculate it: “levodopa equivalent daily dose (LEDD) [15]”. We clarify it in the table. In addition, MNCD" and "EUROHIS-QOL” have been spelt out.**

- English language should be carefully revised as there are many typos.

**AUTHORS – Thank you very much for your comments. English has been reviewed and typos have been corrected. Specifically, it has been reviewed by a worker of Scholar Memory Academic Writing Consultant. We greatly appreciate his help and include it in the acknowledgements.**
